# Clinical study on the effect of multifocal contact lenses on myopia progression in myopia school children: Multifocal contact lens study for suppression of myopia progression

**DOI:** 10.1186/s13063-021-05197-6

**Published:** 2021-03-31

**Authors:** Osamu Hieda, Yo Nakamura, Takahiro Hiraoka, Miho Kojima, Tetsuro Oshika, Chie Sotozono

**Affiliations:** 1 Department of Ophthalmology, Kyoto Prefectural University of Medicine, 465 Kajii-cho, Kawaramachi-Hirokoji, Kamigyo-ku, Kyoto, 602-8566 Japan; 2 Department of Ophthalmology, University of Tsukuba, Tsukuba, Japan

**Keywords:** Multifocal contact lens, Myopia, School children, Suppression of myopia progression

## Abstract

**Background:**

The efficacy of peripheral low add multifocal soft contact lenses (SCLs) for suppressing the progression of myopia is controversial. The aim of the on-going present clinical study is to investigate whether or not multifocal SCLs with + 0.50 diopters (D) addition suppress the progression of myopia in myopic elementary school children.

**Design:**

Prospective randomized controlled trial.

**Subjects and methods:**

The study plans to include a total of 100 myopic school children. Target subjects are primary school male and female students with mild to moderate myopia. Children who have eye-related diseases other than myopia are excluded from the study, because they may affect the evaluation of the outcome. Subjects will be randomly assigned to wear daily disposable multifocal contact lenses with + 0.50D addition or daily disposable SCLs. Subjects will wear contact lenses on both eyes and will be observed for 2 years under a double-masked examination. Primary outcome is a change in the axial length over the 2-year period.

**Objectives:**

The purpose of this study is to identify whether or not multifocal SCLs with + 0.5D addition suppress the progression of myopia in myopic elementary school children as compared with standard SCLs.

**Trial registration:**

1. UMIN (University Hospital Medical Information Network) UMIN000027940. Registered on July 21, 2017

2. JRCT (Japan Registry of Clinical Trials) jRCTs052180172. Registered on March 26, 2019

## Introduction

High incidence of myopia in children is a growing problem in Japan. According to a school health statistics survey conducted in 2018 by the Japanese Ministry of Education, Culture, Sports, Science and Technology, “uncorrected decimal visual acuity (VA) less than 1.0” has been reported in 26.7% of children at kindergartens, 34.2% of children at elementary schools, 56.0% of children at junior high schools, and 67.2% of children at high schools [1]. The survey results further revealed that the VA of elementary school children in 2018 is the worst in recorded history.

Myopia has been more frequently found in lower-grade elementary school children. In general, when myopia develops at a younger age, it tends to progress, thus suggesting an increased incidence rate of high myopia in the future [2]. The increased risk of developing complications such as high myopia-associated retinal detachment, glaucoma, myopic macular degeneration, myopic optic neuropathy, or cataracts is of growing concern.

**Fig. 1:**
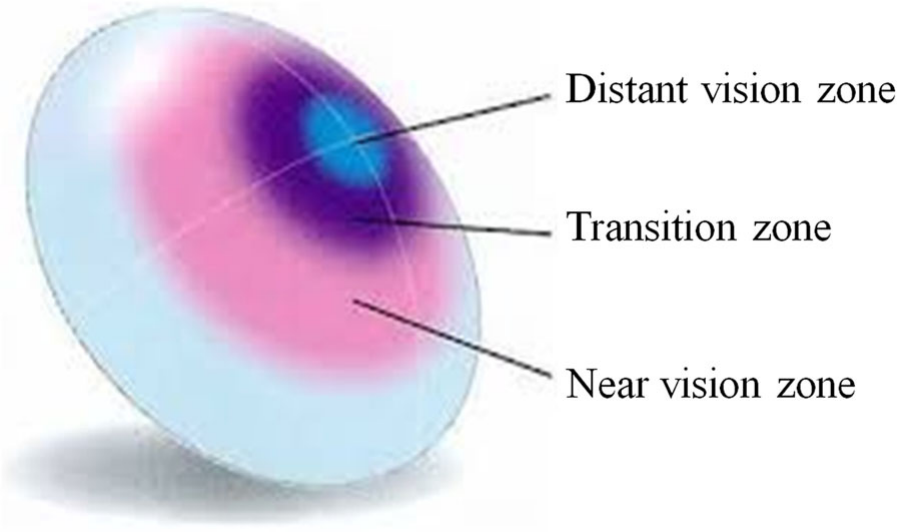
Design of multifocal soft contact lenses (SCLs) with add power in the periphery areas. It is designed as a + 0.5 diopter progressive-add power from the periphery towards the center

If the progression of myopia can be suppressed during the early school years, a time when VA often rapidly decreases, the quality of life in social activities after adolescence can be well maintained. Furthermore, the risk of blindness caused by vison-threatening diseases is expected to be reduced. Therefore, to establish a method to prevent the progression of myopia during the early school years is a critical social issue. For this issue, various therapies such as eye drops [3, 4], progressive addition glasses [5, 6], and orthokeratology lenses [7, 8], etc., have been tested, and a series of research have been done to establish a therapy for better control of the progression of myopia [9]. A recent study has shown that peripheral add multifocal soft contact lenses (SCLs) suppress the progression of myopia (Fig. 1) [10]. However, there are no published studies showing strong evidence that contact lenses with a low add power suppress the progression of myopia.

This study is designed to investigate whether or not multifocal SCLs with a relatively low add power suppress the progression of myopia in school children with highly progressive myopia.

### Primary endpoint

The change of axial length (AL) at the end of the observation period compared to the baseline.

### Secondary endpoints

1. The change of spherical equivalent at each observation time point 0.5, 1, 1.5, and 2 years compared to at baseline.
2. Changes in AL at each observation time point 0.5, 1, and 1.5 years compared to at baseline.
3. Changes in AL and spherical equivalent at each observation time point 0.5, 1, 1.5, and 2 years compared to at the time of enrollment.
4. Observation of the changes in the eyes and extraocular areas by slip-lamp microscopy examination from the start of the study to the end of the observation period (or at the time of study discontinuation).
5. Incidence of adverse events and medical device-related adverse events from the start of the study to the end of the observation period (or at the time of study discontinuation).

### Exploratory endpoints

Subgroup analyses will be performed for patient age, sex, family history, uncorrected distance VA, spherical equivalent power, pupil diameter, lifestyle, and high-order aberrations. These factors could be considered as possible influences on myopia progression.

## Subjects

### Ethical statement

The protocols of this study were approved by the Medical Ethics Review Committee of Kyoto Prefectural University of Medicine, Kyoto, Japan (Approval No. ERB-C-892-2). The study was registered at the University hospital Medical Information Network (UMIN 000027940). After establishing the clinical trials act in Japan, the study was re-approved by the Clinical Research Review Board of Kyoto Prefectural University of Medicine in March 2019 and was registered in the Japan Registry of Clinical Trials (JRCT s052180172). Subjects and their parents/guardians will receive a detailed explanation about the study, and informed consent will be obtained prior to their involvement in the study. The study sponsor is SEED Co., Ltd., yet SEED Co., Ltd. was not involved in any or all of the study design, data collection, management, analysis, and interpretation, the subsequent preparation of the study report, or the decision to submit the final report for publication.

### Study design

This is a prospective therapeutic intervention, multicenter, randomized, double-masked, parallel group comparison study, which will be conducted at the Department of Ophthalmology, Kyoto Prefectural University of Medicine and Department of Ophthalmology, University of Tsukuba, Tsukuba, Japan. The subjects will be independently recruited to compare standard and multifocal SCL (specially controlled medical devices). Authorship was determined with the consent of all authors. This paper will be submitted to the Japanese Journal of Ophthalmology. Since we have no experience with protocol writing, the paper was first written in Japanese and then translated into English. There are no plans for interim and secondary analysis. There are no plans for sharing of the data.

### Subjects

The subjects of this study will be elementary school children with highly progressive mild to moderate myopia. Children who have binocular vision disorder, amblyopia or manifest strabismus, and eye-related diseases other than myopia will be excluded, as they might affect the evaluation of outcomes. Children with any eye disorders in which contact lenses are contraindicated will also be excluded from the study.

#### Inclusion criteria

1. First to sixth grade male and female elementary school students.
2. Children with worsened VA based on the school examination within the previous year compared to that from the previous examinations.
3. Children who can be examined using cycloplegics.
4. Cycloplegic refraction criteria:

- Spherical equivalent power within the range of − 1.00D to − 6.00D in both eyes.
- Difference in the spherical equivalent power between eyes less than 1.50D.
- Astigmatic power within ±1.50D.
- Corrected distance VA with glasses 1.0 or higher.
- Intraocular pressure within the normal range.

5) Children who can visit the study site and receive examinations on the scheduled days.
6) Children and their parents/guardians who provide signed informed consent to participate in the study.

#### Exclusion criteria

Children with any of the following are excluded:

- Binocular vision disorder
- Amblyopia or manifest strabismus
- The difference in the spherical equivalent power with and without cycloplegic 1.50 D or higher in both eyes.
- Eye-related diseases other than myopia
- Systemic diseases that affect vision or refractive error
- Past myopia control therapy, such as bifocal glasses, progressive multifocal lenses, orthokeratology lenses, and drug treatment.
- Children who are determined by investigators to be inappropriate to participate in the study.

### Sample size and randomization

The planned sample size is 50 subjects each at both the Kyoto Prefectural University of Medicine and the University of Tsukuba, i.e., a total of 100 subjects. The sample size is based on the previous published data. Progression of myopia with multifocal SCL and that with standard therapy such as glasses were assumed to be − 0.54D/year and − 0.84D/year, respectively, based on a previous study conducted in USA [11], and standard deviation (SD) was assumed to be 0.66D based on our previous study [12]. Considering that approximately 20% of the subjects would be unevaluable due to study withdrawal, etc., 50 subjects in each group and a total of 100 subjects were determined to be included in this study to detect a between-group significance (alpha = 0.05, two-sided and power = 90%).

Adaptive randomization will be performed to avoid any accidental bias between the two groups. Adaptive stratified sampling will be carried out using the minimization method with genders, grades, and study sites as allocation factors. Randomization is done by an outside clinical research support company. Once consent is obtained, a fax of the case entry is sent and the allocation of the case is sent back.

The meaning of “double-masked” is that both the subjects and the examiners do not know who is in the target group and who is in the control group. An external data center knows whether each case is a subject or a control. The analysis is done in the data center, but in a different department than the allocation.

### Study therapy

**Fig. 2:**
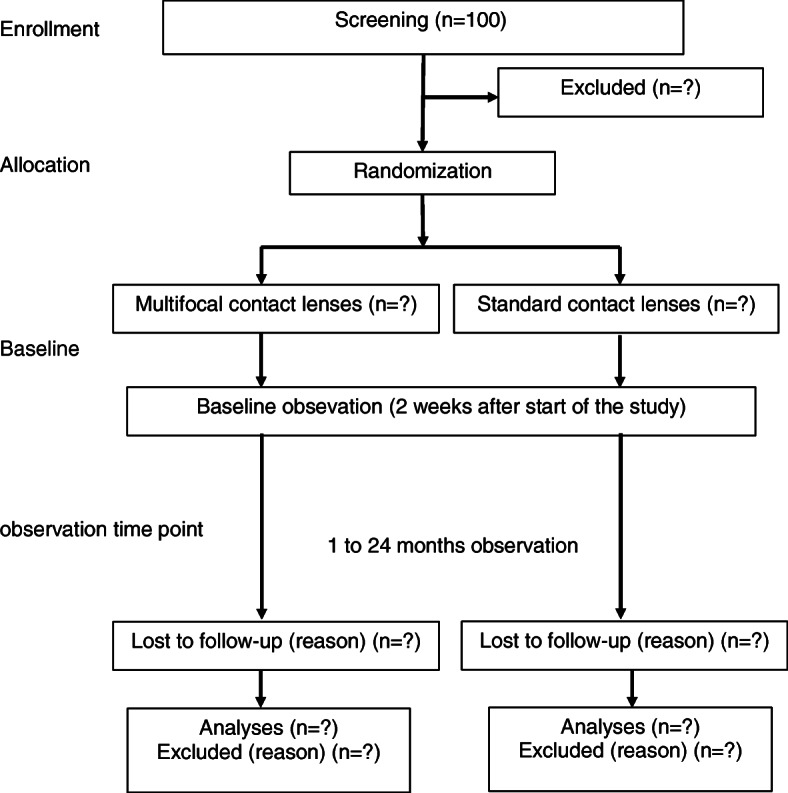
Study flow chart. A same package is used for both multifocal and SCLs, so that subjects and study personnel could not identify the type of SCL

Eligible subjects will be randomly assigned to wear standard SCL SEED 1dayPure UP or SEED 1dayPure UP Flex with + 0.50 D add power in the peripheral areas (SEED Co., Ltd., Tokyo, Japan) (Fig. 1). Contact lens power correction will be determined based on the results of the cycloplegic refraction. Slit-lamp microscopy examination of anterior ocular segment and interviews will be performed 2 weeks and 1, 3, 6, 9, 12, 15, 18, 21, and 24 months after the allocation of the subjects. Cycloplegic refraction, AL, and aberrations (Wavefront sensor, Topcon Corporation, Tokyo, Japan) will be measured 2 weeks and 0.5, 1, 1.5, and 2 years after the allocation. If myopia progresses and the decimal VA with SCL becomes less than 1.0, the contact lenses will be replaced. A flow chart of the study is shown in Fig. 2.

We planned to give the patients only a 3-month supply of contact lenses, and require them to visit the clinic every 3 months. Due to the prevalence of coronavirus, even if hospital follow-up visits do not occur as per the protocol, the contact lenses are being sent to minimize dropouts.

### Outcome measurements

AL was measured using noncontact partial coherence interferometry. The cycloplegic refraction, objective spherical power, astigmatic power and axis, the objective spherical equivalent power, and the mean corneal refractive power will be measured using an auto-refractometer under Cyplegin 1% eye drops. Decimal VA will be examined by orthoptists using the Landolt-C chart. Total higher order, coma-like, spherical-like and spherical aberrations, as well as pupil diameters for cornea/ ocular were measured with or without cycloplegic agents by using the Wavefront sensor KR-1 W®. The difference of each measurement points from the baseline is evaluated.

Wearing conditions of SCL and cornea/conjunctiva will be examined by the slip-lamp microscopy at each follow-up visit. Subjects’ background characteristics will be obtained during the interview at the first visit to the study site. If a serious adverse event occurs, it should be reported to the School Superintendent in accordance with the procedures. Serious adverse events should be listed in the paper. Medical interviews and slit-lamp microscopy examinations will be conducted at each hospital visit to identify adverse events. Clinically important adverse events will be described in the paper. If any health damage occurs in relation to this clinical research, it will be compensated by the clinical research insurance purchased for this clinical research.

### Statistical analyses

All data will be analyzed based on the “intention-to-treat” analysis. The primary analysis will be performed in the full analysis set (FAS), and robustness of the results will be explored through sensitivity analysis in the per-protocol set (PPS). The full analysis set (FAS) refers to all subjects who consented to start wearing contacts, and the PPS refers to subjects who were able to wear contacts on schedule for 2 years. No interim analysis is planned.

#### Data management

INCREASE Co., Ltd., Tokyo, Japan, will act as a data center, and manage and handle study data that include data cleaning.

#### Data analyses

Summary statistics will be calculated for test values and change values at the end of the observation period (24 months) from the baseline (2 weeks). Between-group comparisons will be performed using the mixed effects model for repeated measures in both eyes, and individuals as random effects. The significance level is set at 5% (two-sided). All statistical analyses were performed using SAS version 9.4 (SAS Institute Inc., Cary, NC, USA) software.

### Data monitoring and audits

Contracted monitoring organization is INCREASE Co., Ltd. The data center will perform the central data monitoring using the electronic data of the case report forms. Central monitoring will identify missing values and ask each facility to make up for them. If it is truly deficient, it was noted. Data monitoring at study sites will also be performed by using sampled subjects. Results will be reported after monitoring. If any data is missing, the missing values are to be complemented by the single-assignment method.

Contracted auditing organization is INCREASE Co., Ltd. Results will be reported after audit.

## Discussion

Previous studies show that three types of SCL are effective for suppressing the progression of myopia, namely, concentric bifocal lenses [13], extended depth of focus lenses [14], and peripheral add multifocal SCL. The first two have been certified as a CE mark for suppressing the progression of myopia in Europe.

Of peripheral add multifocal SCL, the effectiveness of those with + 2D higher power add has been reported in previous studies [11, 15]. However, they might be unsuitable for elementary school children because of the visual instability. Low add multifocal SCL are commercially available to prevent eyestrain, and they do not lead to the development of presbyopia symptoms such as eye fatigue, eye pain, headache, and so on. The impact of wearing SCL in young children is not yet fully understood.

The potential mechanisms of how the low add multifocal SCL suppress the progression of myopia include a prevention of hyperopic blur on the peripheral retina [16], and decreased accommodation stimuli by the increased multifocality; however, their actions are not yet fully understood in detail. Fujikado et al. shown a significant effect of low add SCL on the AL change but not on the refractive error [17]. Our study is multicenter with expanded number of subjects and longer observation period, to decrease bias. If the present double-masked study confirms significant changes in both AL and refractive error in the low add multifocal SCL group, they might become a standard therapy for myopia correction in school-aged children in Japan.

## Data Availability

Data sharing is not applicable to this article as no datasets were generated or analyzed during the current study.

## Abbreviations

AL: Axial length
D: Diopters
SCL: Soft contact lens
VA: Visual acuity
